# Supplementary material for: Genome-wide association analysis and Mendelian randomization proteomics identify drug targets for heart failure
Source: Nat Commun. 2023 Jul 10;14:3826. doi: 10.1038/s41467-023-39253-3 (PMC10333277; doi:10.1038/s41467-023-39253-3)
Supplement: Supplementary file 3 — Description of Additional Supplementary Files [file 41467_2023_39253_MOESM3_ESM.pdf]

Description of Additional Supplementary Files

File Name: Supplementary Data 1
Description: Clinical and demographic characteristics of included participants in the heart failure meta-analysis.

File Name: Supplementary Data 2
Description: Previously reported heart failure GWAS variants. All tests were two-sided without adjustment for multiple testing.

File Name: Supplementary Data 3
Description: Genetic associations of 18 novel loci in heart failure risk factors and left ventricle cardiac MRI traits. All tests were two-sided without adjustment for multiple testing.

File Name: Supplementary Data 4
Description: Participant characteristics from Million Veteran Program (MVP) African-American cohort

File Name: Supplementary Data 5
Description: Genetic associations of 39 GWAS-associated loci in African-American MVP cohort. All tests were two-sided without adjustment for multiple testing.

File Name: Supplementary Data 6
Description: Colocalization results for heart failure. Presented for results that pass MR *P*-value < 0.0001.

File Name: Supplementary Data 7
Description: Mendelian randomization results for heart failure risk factors and left ventricle cardiac MRI traits using cis-pQTL instruments (SomaLogic platform from Fenland study). All tests were two-sided without adjustment for multiple testing.

File Name: Supplementary Data 8
Description: Colocalization results for HF risk factors and left ventricle cardiac MRI GWAS and cis-pQTL GWAS. Presented for results that pass MR *P*-value < 0.0001.

File Name: Supplementary Data 9
Description: cis-eQTL associations identified in eQTL-Gen and GTEx V8 for 18 loci identified from 10 MR-Proteomics heart failure-associated genes. All tests were two-sided without adjustment for multiple testing.

File Name: Supplementary Data 10
Description: Secondary biological hits identified for horizontal pleiotropy analysis using gene expression from eQTLGen and proteins from SOMAScan V4 from Fenland Study. All tests were two-sided without adjustment for multiple testing.

File Name: Supplementary Data 11
Description: Genetic correlation of heart failure with other cardiovascular traits. All tests were two-sided and adjusted for multiple comparisons.

File Name: Supplementary Data 12
Description: Number of cis-eQTLs per GTEx V8 tissue from previously published and newly identified heart failure genome-wide significant loci (n=40) with 18 proposed instruments from 10 MR-Proteomics genes associated with heart failure.

File Name: Supplementary Data 13
Description: Significantly enriched biological pathways from eQTLs associated with previously published and newly identified heart failure genome-wide significant loci (n=40) and 18 proposed instruments from 10 MR-Proteomics heart failure-associated genes. All tests were two-sided and adjusted for multiple comparisons.

File Name: Supplementary Data 14
Description: Enriched biological pathways over representation analysis counts for 18 newly identified heart failure GWAS-associated loci. All tests were two-sided and adjusted for multiple comparisons.

File Name: Supplementary Data 15
Description: EpiGraphDB results.

File Name: Supplementary Data 16
Description: Animal knockout models.

File Name: Supplementary Data 17
Description: Drug or chemical compound and mechanism of action extracted from ChEMBL for identified druggable genes

File Name: Supplementary Data 18
Description: In-silico trials on 18 GWAS-associated heart failure loci and 10 MR-Proteomics identified heart failure genes. All tests were two-sided without adjustment for multiple comparisons.

File Name: Supplementary Data 19
Description: Comparison with Global Biobank Meta-analysis Initiative (GBMI) analysis on heart failure. All tests were two-sided without adjustment for multiple comparisons.

File Name: Supplementary Data 20
Description: Replication of 18 novel loci in GBMI meta-analysis without UK Biobank. All tests were two-sided without adjustment for multiple comparisons.

File Name: Supplementary Data 21

Description: Population characteristics of polygenic risk score (PRS) testing cohort in the BioVU. Cases and controls characteristics compared using two-sample t-test and results are significant at $p < 0.05$.
