# Supplementary material for: Genome-wide association analysis and Mendelian randomization proteomics identify drug targets for heart failure
Source: Nat Commun. 2023 Jul 10;14:3826. doi: 10.1038/s41467-023-39253-3 (PMC10333277; doi:10.1038/s41467-023-39253-3)
Supplement: Supplementary file 5 — Reporting Summary [file 41467_2023_39253_MOESM5_ESM.pdf]

nature portfolio

Corresponding author(s): Dr. Danielle Rasooly

Last updated by author(s): 2023/05/15

Reporting Summary

Nature Portfolio wishes to improve the reproducibility of the work that we publish. This form provides structure for consistency and transparency in reporting. For further information on Nature Portfolio policies, see our Editorial Policies and the Editorial Policy Checklist.

Statistics

For all statistical analyses, confirm that the following items are present in the figure legend, table legend, main text, or Methods section.

n/a	Confirmed	
☐	☒	The exact sample size (*n*) for each experimental group/condition, given as a discrete number and unit of measurement
☒	☐	A statement on whether measurements were taken from distinct samples or whether the same sample was measured repeatedly
☐	☒	The statistical test(s) used AND whether they are one- or two-sided<br>*Only common tests should be described solely by name; describe more complex techniques in the Methods section.*
☐	☒	A description of all covariates tested
☐	☒	A description of any assumptions or corrections, such as tests of normality and adjustment for multiple comparisons
☐	☒	A full description of the statistical parameters including central tendency (e.g. means) or other basic estimates (e.g. regression coefficient) AND variation (e.g. standard deviation) or associated estimates of uncertainty (e.g. confidence intervals)
☐	☒	For null hypothesis testing, the test statistic (e.g. *F*, *t*, *r*) with confidence intervals, effect sizes, degrees of freedom and *P* value noted<br>*Give P values as exact values whenever suitable.*
☐	☒	For Bayesian analysis, information on the choice of priors and Markov chain Monte Carlo settings
☒	☐	For hierarchical and complex designs, identification of the appropriate level for tests and full reporting of outcomes
☒	☐	Estimates of effect sizes (e.g. Cohen's *d*, Pearson's *r*), indicating how they were calculated

Our web collection on statistics for biologists contains articles on many of the points above.

Software and code

Policy information about availability of computer code

Data collection	Meta-analysis of GWAS summary statistics were prepared using publicly available software, including METAL (https://genome.sph.umich.edu/wiki/METAL_Documentation), version release 2020-05-05. Pre-phasing of the MVP data was executed using the EAGLE Version 2.4 software, and imputation was executed using the 1000 Genomes Phase 3 reference panel using Minimac4. Phasing of the HERMES Consortium data was conducted using Eagle, MaCH, and SHAPEIT software, and imputation was conducted using mimimac2 and IMPUTE2. Software used to annotate our results are described in the Methods section of the manuscript.
Data analysis	We used publicly available software for the analyses, and all software used is listed and described in the Methods section of our manuscript. Statistical analyses were conducted in R version 3.6.3. Mendelian randomization analyses were conducted using the TwoSampleMR package in R version 0.5.3 (https://mrcieu.github.io/TwoSampleMR/), genetic colocalization analyses were conducted using the coloc package in R (https://cran.r-project.org/web/packages/coloc/index.html and https://chr1swallace.github.io/coloc, using default priors), pathway enrichment analyses were conducted using the clusterProfiler package in R (https://pubmed.ncbi.nlm.nih.gov/22455463/) and the enrichplot R package, LD Score regression was conducting using LDSC (https://github.com/bulik/ldsc), and polygenic risk score was calculated using the PRS-cs package v1.0.0 (https://github.com/getian107/PRScs).

For manuscripts utilizing custom algorithms or software that are central to the research but not yet described in published literature, software must be made available to editors and reviewers. We strongly encourage code deposition in a community repository (e.g. GitHub). See the Nature Portfolio guidelines for submitting code & software for further information.

Data

Policy information about availability of data

All manuscripts must include a data availability statement. This statement should provide the following information, where applicable:

- Accession codes, unique identifiers, or web links for publicly available datasets
- A description of any restrictions on data availability
- For clinical datasets or third party data, please ensure that the statement adheres to our policy

The MVP GWAS summary statistics used in this study is available through dbGAP under accession code phs001672.v10 [https://www.ncbi.nlm.nih.gov/projects/gap/cgi-bin/study.cgi?study_id=phs001672.v10.p1]. The only restriction is that use of the data is limited to health/medical/biomedical purposes, and does not include the study of population origins or ancestry. Use of the data does include methods development research (e.g., development and testing of software or algorithms) and requesters agree to make the results of studies using the data available to the larger scientific community. The HERMES GWAS summary statistics used in this study are publicly available at the GWAS Catalog under accession code GCST009541 [https://www.ebi.ac.uk/gwas/studies/GCST009541]. Fenland-SomaLogic protein GWAS data are available at https://omicscience.org/. GTEx project v.8 data are publicly available at https://gtexportal.org/home/. Mouse Genome Informatics (MGI) data is publicly available at http://www.informatics.jax.org/. The GWAS summary statistics for the risk factor analyses used in this study are deposited in the GWAS Catalog (https://www.ebi.ac.uk/gwas/) and the accession codes are as follows: body mass index (GCST006900), alcohol consumption (GCST007325), atrial fibrillation (GCST006414), systolic blood pressure (GCST006624), diastolic blood pressure (GCST006630), type 2 diabetes (GCST006867), and coronary artery disease (GCST005194) troponin (GCST005806), NT-pBNP (GCST005806) and IL-6 (GCST90012049). The GWAS summary statistics for smoking and chronic obstructive airways disease used in this study are available at https://gwas.mrcieu.ac.uk under GWAS ID ukb-b-5779 and ukb-b-13447, respectively, and the GWAS summary statistics for the traits examined in the in-silico trails are available at https://gwas.mrcieu.ac.uk using the GWAS IDs listed in the Supplementary Table. The GWAS summary statistics for the LDL-cholesterol and HDL-cholesterol are publicly available in http://csg.sph.umich.edu/willer/public/glgc-lipids2021/results/ancestry_specific/. The summary statistics for estimated glomerular filtration rate (eGFR) are deposited in ://www.uni-regensburg.de/medizin/epidemiologie-praeventivmedizin/genetische-epidemiologie/gwas-summary-statistics/index.html. The cardiac MRI datasets provided by Pirruccello et al are deposited under Dataset Name "UK Biobank Cardiac MRI LV GWAS" on https://cvd.hugeamp.org/downloads.html. The Open Targets data are deposited in https://platform.opentargets.org/. The EpiGraphDB database used in this study is provided at: https://www.epigraphdb.org/.

Research involving human participants, their data, or biological material

Policy information about studies with human participants or human data. See also policy information about sex, gender (identity/presentation), and sexual orientation and race, ethnicity and racism.

Reporting on sex and gender	Findings apply to both sexes and only sex was used in this analysis. The information on the sex of the participants was obtained from the provided phenotype information by the HERMES Consortium and the MVP. The MVP participants included 96.92% and 92.14% males for cases and controls, respectively, and the HERMES Consortium participants included 59.13% and 47.15% males for cases and controls, respectively.
Reporting on race, ethnicity, or other socially relevant groupings	The findings presented in this study apply to individuals of European ancestry, and this information was determined and provided from the phenotype data by the HERMES Consortium and the MVP.
Population characteristics	The HERMES GWAS comprised of 47,309 cases and 930,014 controls of European ancestry across 26 studies from the Heart Failure Molecular Epidemiology for Therapeutic Targets (HERMES) Consortium, where the study sample included both population cohorts (17 studies, 38,780 HF cases, 893,657 controls) and case-control samples (9 studies, 8,529 cases, 36,357 controls) and cases were identified with a clinical diagnosis of HF of any aetiology with no inclusion criteria based on LV ejection fraction and controls were participants without HF. The characteristics of each participating studies, including age, body mass index, sex, and other heart failure risk factors, are presented in Supplementary Data 12 of the manuscript at PMID: 31919418. The HERMES Consortium participants were 59.13% male for cases and 47.15% male for controls, with average age (years) of 69.40 (SD 4.17) for cases and 62.16 (SD 10.63) for controls. The MVP participants (n=302,258) were predominantly male (92.1% for controls and 96.9% for cases), and the average age at study enrollment was 62.7 for controls and 69.6 for cases. Mean body mass index (BMI) was 29.2 (SD: 5.5) and 31.1 for controls and cases, respectively.
Recruitment	Veterans aged 19 to 104 years were voluntarily recruited from more than 63 Veterans Health Administration Medical Centers nationwide since 2011 for participation in the Million Veteran Program Biobank study. The design of the Million Veteran Program along with a complete description of the recruitment has been described previously (PMID: 26441289). A description of the recruitment along with dates for the recruitment period for each study included in the HERMES consortium can be found in the cohort descriptions Supplementary File of the manuscript (PMID: 34480422).
Ethics oversight	All included studies in the HERMES consortium were ethically approved by local institutional review boards and all participants provided written informed consent. The MVP received ethical and study protocol approval by the Veterans Affairs Central Institutional Review Board and informed consent was obtained for all participants. The Fenland study was approved by the National Health Service (NHS) Health Research Authority Research Ethics Committee (NRES Committee – East of England Cambridge Central, ref. 04/Q0108/19), and all participants provided written informed consent.

Note that full information on the approval of the study protocol must also be provided in the manuscript.

Field-specific reporting

Please select the one below that is the best fit for your research. If you are not sure, read the appropriate sections before making your selection.

☒ Life sciences ☐ Behavioural & social sciences ☐ Ecological, evolutionary & environmental sciences

For a reference copy of the document with all sections, see nature.com/documents/nr-reporting-summary-flat.pdf

Life sciences study design

All studies must disclose on these points even when the disclosure is negative.

Sample size	The sample size of the GWAS meta-analysis of HERMES and MVP heart failure GWAS datasets was determined based on using all genetic data available from MVP/HERMES.
Data exclusions	In the meta-analysis of heart failure from the published MVP and HERMES GWAS, we removed variants with a MAF < 0.5%. Further, we restricted instrumental variables to conditionally independent variants that act locally, or are located in cis, to reduce the chances of horizontal pleiotropy.
Replication	The meta-analysis framework includes replication by default because it weights the reported statistics identified in each study by the evidence of association across the multiple studies. For the heart failure results that passed our significance thresholds for Mendelian randomization and colocalization analyses, we conducted Mendelian randomization and colocalization analyses against 15 heart failure risk factors and 9 left ventricular cardiac MRI traits. These were performed independently, and the results of the replication per variant and per trait are reported in the manuscript. Further, we carried out SNP-based replication for the novel loci by comparing our findings to those found in an independent sample by the Global Biobank Meta-analysis Initiative (GBMI). This replication was performed independently, and the results of the replication per variant are reported in the Supplementary Data. Findings from the GBMI replication study of the 18 novel loci identified 100% of the variants have a beta estimate that is directionally concordant with our meta-analysis and 61.1% (11 of 18) that are nominally significant (p-value < 0.05).
Randomization	Randomization is not applicable, as this is an observational study and did not randomly allocate individuals to a particular intervention.
Blinding	Blinding is not applicable, as this is an observational study and did not require blinding to a treatment status.

Reporting for specific materials, systems and methods

We require information from authors about some types of materials, experimental systems and methods used in many studies. Here, indicate whether each material, system or method listed is relevant to your study. If you are not sure if a list item applies to your research, read the appropriate section before selecting a response.

Materials & experimental systems

n/a	Involved in the study	
☒	☐	Antibodies
☒	☐	Eukaryotic cell lines
☒	☐	Palaeontology and archaeology
☒	☐	Animals and other organisms
☒	☐	Clinical data
☒	☐	Dual use research of concern
☒	☐	Plants

Methods

n/a	Involved in the study	
☒	☐	ChIP-seq
☒	☐	Flow cytometry
☒	☐	MRI-based neuroimaging
